# Lipidomic Assessment of the Inhibitory Effect of Standardized Water Extract of *Hydrangea serrata* (Thunb.) Ser. Leaves during Adipogenesis

**DOI:** 10.3390/nu16101508

**Published:** 2024-05-16

**Authors:** Jae Sik Yu, Hee Ju Kim, Yeo Eun Kim, Hyun Ok Yang, Yu-Kyong Shin, Hyunjae Kim, Soyoon Park, Gakyung Lee

**Affiliations:** 1 Department of Integrative Biological Sciences and Industry, Sejong University, Seoul 05006, Republic of Korea; jsyu@sejong.ac.kr (J.S.Y.); heeju110609@naver.com (H.J.K.); ajh0485@naver.com (Y.E.K.); hoyang@sejong.ac.kr (H.O.Y.); 2 Convergence Research Center for Natural Products, Sejong University, Seoul 05006, Republic of Korea; 3 Department of New Material Development, COSMAXBIO, Seongnam 13486, Republic of Korea; ykshin@cosmax.com (Y.-K.S.); hyounjeakim@cosmax.com (H.K.); soyoon.park@cosmax.com (S.P.)

**Keywords:** *Hydrangea serrata*, adipogenesis, lipidomics, anti-obesity, lipid metabolism

## Abstract

Obesity is primarily exacerbated by excessive lipid accumulation during adipogenesis, with triacylglycerol (TG) as a major lipid marker. However, as the association between numerous lipid markers and various health conditions has recently been revealed, investigating the lipid metabolism in detail has become necessary. This study investigates the lipid metabolic effects of *Hydrangea serrata* (Thunb.) Ser. hot water leaf extract (WHS) on adipogenesis using LC-MS-based lipidomics analysis of undifferentiated, differentiated, and WHS-treated differentiated 3T3-L1 cells. WHS treatment effectively suppressed the elevation of glycerolipids, including TG and DG, and prevented a molecular shift in fatty acyl composition towards long-chain unsaturated fatty acids. This shift also impacted glycerophospholipid metabolism. Additionally, WHS stabilized significant lipid markers such as the PC/PE and LPC/PE ratios, SM, and Cer, which are associated with obesity and related comorbidities. This study suggests that WHS could reduce obesity-related risk factors by regulating lipid markers during adipogenesis. This study is the first to assess the underlying lipidomic mechanisms of the adipogenesis-inhibitory effect of WHS, highlighting its potential in developing natural products for treating obesity and related conditions. Our study provides a new strategy for the development of natural products for the treatment of obesity and related diseases.

## 1. Introduction

Obesity is an increasing global health threat, acting as a critical risk factor for the onset of comorbidities such as diabetes, fatty liver, and cardiovascular disease [1,2,3]. A key contributor to obesity is lipid accumulation, primarily initiated through excessive lipogenesis [4]. This process begins when adipogenesis is triggered by the activation of specific regulators during preadipocyte differentiation, leading to the storage of lipids within cells [5]. A notable metabolic shift during the adipogenesis is the elevation of triacylgycerol (TG) levels [6]. Although total TG levels are a conventional marker in obesity management, the diversity among hundreds of TG species, each consisting of glycerol bonded to three distinct fatty acyl chains, plays a significant role due to their varied biological functions [7,8]. Furthermore, it has been known that alterations in certain lipid species and compositional changes in the metabolism of lipids other than TG, such as sphingolipids and glycerophospholipids occurring during adipogenesis, are also closely correlated with obesity-related diseases and metabolic disorders [9,10]. Lipid metabolism plays a variety of roles in the process of obesity and adipogenesis, but comprehensive research of the inhibitory effect on adipogenesis has focused only on measuring total TG and cholesterol. Therefore, investigating the regulation mechanisms of overall lipid metabolism, including composition changes, holds great significance to clarify the effect of substances targeting adipogenesis inhibition. This approach not only aids in identifying lipid markers for the comorbidities associated with the substance but also enhances understanding of its relationship with related diseases.

*Hydrangea serrata* (Thunb.) Ser. (*H. serrata*), is an indigenous plant species in Korea and is commonly known as ‘Mountain Hydrangea [11]. Its leaves are consumed as herbal tea in Korea and other Asian countries [12]. The extract of *H. serrata* leaves has been reported to have various health benefits, including anti-photoaging and a hepatoprotective effect [13,14,15]. Specifically, a hot water extract from the leaves of the *H. serrata* (WHS) has a strong effect against obesity in db/db and high fat diet-induced mice, and eventually in human clinical trials as well without any safety issues over 12 weeks of repeated administration. [16,17,18]. WHS effectively regulates an AMP-activated protein kinase and inhibits the gene expression of adipogenic transcription factor: CCAAT/enhancer binding protein (C/EBP) α, peroxisome proliferator-activated receptor (PPAR) γ, and sterol regulatory element binding protein (SREBP)-1C. Despite the several studies, the results related to lipid metabolism represented with only total LDL, HDL, cholesterol, and TG through biochemical examination of blood. In-depth perspectives of the responsible lipid metabolites for these changes were not investigated. Therefore, unlike previous approaches, the investigation of lipid profiles on the inhibitory effects of adipogenesis effected by WHS may provide new insights through specific mechanisms of lipid metabolism regulation that remain unexplained to date.

Lipidomics, a branch of metabolomics that focuses on the comprehensive analysis of lipids in biological systems, is an important tool for understanding the complex processes of cellular metabolism [19]. This technique has been widely used in biomedical research to evaluate the toxicological and pharmacological properties of drugs, to understand pathological mechanisms for disease states, and to discover potential biomarkers [20]. In particular, the lipidomic approach in adipogenesis, which is closely related to lipid metabolism [9], can provide crucial insights to explore the therapeutic potential of medicinal plants as effective regulators of adipogenesis.

This study aims to evaluate the effect of treatment with standardized WHS on lipid profile during adipogenesis in 3T3-L1 cells, thereby identifying the regulation of lipid accumulation and key adipogenic markers. This provides a scientific basis for potential treatments for obesity and metabolic disorders and expands the understanding of the metabolic mechanisms of natural products in modulating adipogenesis.

## 2. Materials and Methods

### 2.1. Preparation of Water Extract of Hydrangea serrata (Thunb.) Ser. Leaves (WHS)

WHS was prepared and standardized as previous described [14]. Briefly, *Hydrangea serrata* (Thunb.) Ser. was verified by Dr. Hyung-Jun Kim from Forest Medicinal Resources Research Center in National Institute of Forest Science (NIFoS, Seoul, Republic of Korea) and a voucher specimen was deposited in COSMAXBIO (COSMAXBIO, Seongnam, Republic of Korea). The dried leaves of *H. serrata* (1000 g) were extracted with distilled water at 98 °C for 5 h, followed by filtration, and then they were spray dried.

### 2.2. 3T3-L1 Cell Culture and Differentiation

**Figure 1:**
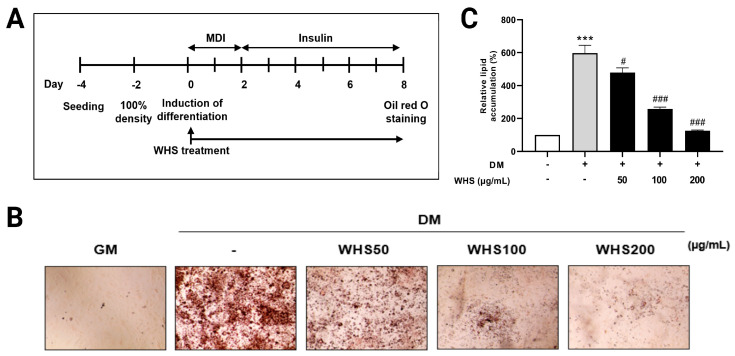
Effect of WHS on the differentiation of 3T3-L1 preadipocytes. 3T3-L1 preadipocytes were differentiated with or without WHS treatment (50, 100, 200 μg/mL) for 8 days. (**A**) Schematic of the differentiation process of 3T3-L1 cells and WHS treatment. (**B**) Lipid content in 3T3-L1 cells assessed by Oil-Red O staining. (**C**) Calculated relative lipid content in 3T3-L1 cells. Cells were treated with isopropanol, and lipid accumulation was measured by absorbance at OD 500 nm. GM denotes the growth media group (control); DM denotes the differentiation media group (differentiation); WHS denotes the differentiated group treated with WHS at 50, 100, 200 μg/mL. Graphs are presented as mean ± SEM (*n* = 5, *** *p* < 0.0001 vs. GM; # *p* < 0.05 vs. DM; ### *p* < 0.001 vs. DM).

The 3T3-L1 preadipocyte cells were purchased from the American Type Culture Collection (Manassas, VA, USA). The 3T3-L1 cells (3 × 10^5^/well) were seeded on 6-well plates and adipocyte differentiation was induced for 8 days with WHS (Figure 1A). The cells were cultured in DMEM supplemented with 10% Newborn Calf Serum (NCS), 1% penicillin/streptomycin (Gibco, Grand Island, NY, USA) at 37 °C with 5% CO_2_. For differentiation, 3T3-L1 cells were treated with DMEM (Gibco, Grand Island, NY, USA) supplemented with 10% Fetal Bovine Serum (FBS), 1% penicillin/streptomycin (Gibco, Grand Island, NY, USA) medium containing 5 mM 3-Isobutyl-1-methylxanthine (IBMX), 1 μM dexamethasone, and 10 μg/mL insulin (MDI), with or without WHS. After 2 days, the differentiation medium containing MDI was aspirated and replaced with fresh culture medium supplemented with 10 μg/mL insulin, continuing with or without WHS. This process was repeated every 2 days until the 8th day. These cell experiments were conducted using five biological replicates.

### 2.3. Oil-Red O Staining

On the 8th day, cells were washed with phosphate-buffered saline (PBS) and fixed with 4% paraformaldehyde for 1 h in a cold room. After discarding paraformaldehyde, Oil-red O solution (Sigma Aldrich, St. Louis, MO, USA) was added to each well and left at room temperature for 30 min. Then, cells were washed with distilled water and imaged under a microscope (EVOS™XL Core, Invitrogen, Waltham, MA, USA). Oil-red O in lipid droplets was extracted with 100% isopropanol and measured using a microplate reader at OD 500 nm (Multiskan SkyHigh, Thermo Scientific, Waltham, MA, USA). Measurements were performed in triplicate for each sample, and the average value was calculated for use in statistical analysis.

### 2.4. Lipid Extraction

Cells were collected by removing the media and washing with PBS. A total of 400 µL of CHCl_3_/MeOH solution (2:1 *v*/*v*) containing internal standards mixture (SPLASH II LIPIDOMIX Mass Spec Standard, Avanti Polar Lipids, Alabaster, AL, USA) was added to the cell pellet. The mixture was vortexed for 1 min and incubated for 1 h on ice. Then, 150 µL of DW was added to sample, vortexed and stood on ice for additional 10 min. Samples were centrifuged at 14,000 rpm, 4 °C for 5 min and the lower phase (organic) layer was transferred to a new tube. The lower phase was dried using a centrifugal vacuum concentrator and reconstituted in 120 μL of isopropanol. Finally, 5 μL of the sample was injected for lipidomic analysis.

### 2.5. Lipidomic Analysis and Data Processing

The prepared samples underwent analysis using an Orbitrap Exploris™ 120 Mass spectrometer coupled with Vanquish Flex UHPLC system (Thermo Fisher Scientific, San Jose, CA, USA) at the Biopolymer Research Center for Advanced Materials (Sejong University, Seoul, Republic of Korea). Samples of 5 μL were injected in a randomized order within an analytical batch. Quality control (QC) samples which were made by mixing equal volume from the set of samples were analyzed every ten samples throughout the batch to ensure consistency and reliability of the analytical process. For chromatographic separations, an ACQUITY UPLC^®^ BEH C18 column (2.1 × 100 mm, 1.7 µm, Waters, Milford, MA, USA) with 40% acetonitrile (*v*/*v*, mobile phase A) and isopropanol/acetonitrile (90:10, *v*/*v*, mobile phase B), both containing 2 mM ammonium formate and 0.1% formic acid, was used. The gradient conditions were as follows: 0–1 min, 10% B; 1–6 min, 10–70% B; 6–12 min, 70–90% B; 12–13 min, 90–100% B; 13–13.5 min, 100–10% B; 13.5–16 min, 10% B for re-equilibrium. The flow rate was 0.35 mL/min and the column temperature was 45 °C. The compounds were detected with a full scan range (m/z 80–1200) at a resolution of 120,000. The detailed parameters were as follows: sheath gas flow, 50 arb; auxiliary gas flow, 10 arb; sweep gas flow, 1 arb; spray voltage, 3500 V; ion transfer tube temperature, 325 °C; vaporizer temperature, 350 °C and S-lens RF level, 70%. The data-dependent MS2 scan was performed. All of the UPLC-Orbitrap-MS data were acquired using Xcalibur 4.6 and preprocessed using Compound Discoverer 3.3 (Thermo Fisher Scientific). The extracted and aligned spectra were identified with by MS2 fragmentation matching with a 5 ppm mass tolerance.

### 2.6. Statistical Analysis

The results of Oil-Red O staining were analyzed with Prism 9.0 software (GraphPad Software, Inc., San Diego, CA, USA) using one-way ANOVA. For lipidomic study, the intensities of identified lipids were normalized by the peak area of an internal standard (IS) corresponding to each class. SIMCA 14.1 (Umetrics, Inc., Ume, Sweden) was used to perform the multivariate analysis and Wilcoxon rank-sum test was conducted to assess group difference between GM and DM group, and DM- and WHS-treated groups using R Studio (https://www.r-project.org/ (accessed on 26 October 2023)). To discover the correlation between lipid changes and pattern of fatty acyl chain (carbon chain length and double bond), Spearman correlation test was performed. Data were expressed as the means ± standard error of the mean (SEM) and *p*-value < 0.05 were considered statistically significant.

## 3. Results and Discussion

### 3.1. WHS Inhibits the Lipid Accumulation during Adipogenesis in 3T3-L1 Preadipocytes

The inhibitory effects of WHS on body fat accumulation and adipocyte hypertrophy in HFD-induced obese mice have been confirmed in previous studies [17]. However, the underlying mechanisms of lipid metabolism remain unclear. To investigate these mechanisms, we first verified the inhibitory effect of WHS on lipid accumulation during adipogenesis and then conducted lipidomic analysis on the treated cell samples. The 3T3-L1 cells were differentiated with various treated concentrations of WHS (50, 100, 200 µg/mL) for 8 days (Figure 1A). Lipid accumulation was measured by Oil-Red O staining. As shown in Figure 1B, WHS inhibited the accumulation of lipid droplets in a dependent manner. The observed values of extracted Oil-Red O in 3T3-L1 adipocytes represent the lipid droplet accumulation in the cytoplasm of 3T3-L1 adipocyte cells. (Figure 1C), WHS treatment significantly reduced the absorbance value of the extracted Oil-Red O solution, when compared to the differentiated adipocyte in the without of WHS (*p* < 0.0001). These results suggest that WHS effectively inhibited fat accumulation in 3T3-L1 adipocytes and indicate that WHS could influence lipid metabolism related to the adipogenesis process, such as TG.

### 3.2. Difference in Lipid Profile of Adipogenesis in 3T3-L1 Preadipocytes Cell and WHS Treatment

**Figure 2:**
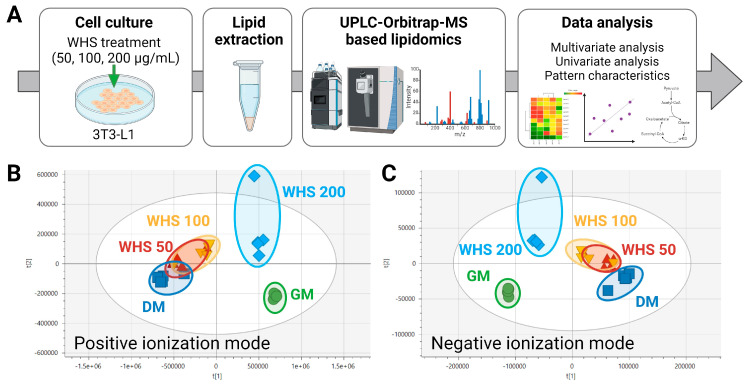
Lipid profiling in 3T3-L1 cells. (**A**) Experimental design: GM, DM, and DM with WHS treatment at various concentrations (50, 100, 200 μg/mL); cells were harvested, and lipids extracted; lipidomic analysis was conducted using UPLC-Orbitrap-MS; data processing and statistical analyses of the obtained lipid profiles were performed. Principal component analysis (PCA) score plots derived from the lipidomics data in 3T3-L1 cells in (**B**) positive and (**C**) negative ionization mode. GM denotes the growth media group (control); DM denotes the differentiation media group (differentiation); WHS denotes the differentiated group treated with WHS at 50, 100, 200 μg/mL.

**Figure 3:**
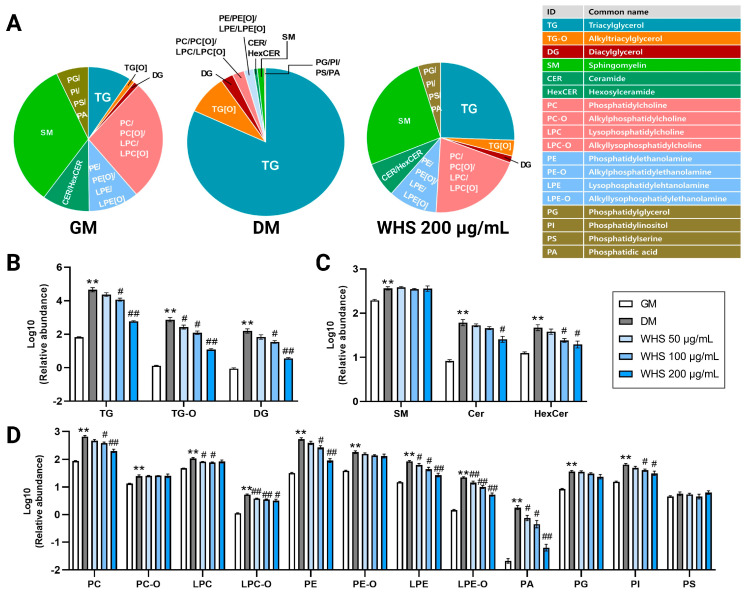
Overview of the effects of WHS treatment on the cell lipidome during the differentiation of 3T3-L1 cells. (**A**) The cellular lipid composition of GM, DM, and WHS-treated (200 μg/mL) groups. The total lipid abundance of (**B**) Glycerolipids, (**C**) Sphingolipids, and (**D**) Glycerophospholipids. GM denotes the growth media group (control); DM denotes the differentiation media group (differentiation); WHS denotes the differentiated group treated with WHS at 50, 100, 200 μg/mL. The data are expressed as log10 of total relative abundance of identified lipids. Graphs are presented as mean ± SEM (*n* = 5, ** *p* < 0.01 vs. GM; # *p* < 0.05 vs. DM; ## *p* < 0.01 vs. DM). The lipid subclasses are indicated on the *x*-axis.

To explore significant differences in the lipidomic profiles among the groups, the lipid extract from 3T3-L1 cell were analyzed using in-house UPLC-Orbitrap-MS-based lipidomics method (Figure 2A). A total of 621 lipids were identified in the 3T3-L1 cells through the lipidomic analysis and these included 191 Glycerolipids (GLs), 343 Glycerophospholipids, and 85 Sphingolipids. These 3 classes of lipids were classified into 18 classes included triacylglycerol (TG), alkyltriacylglycerol (TG-O), diacylglycerol (DG), sphingomyelin (SM), ceramides (Cer), Hexosylceramide (HexCer), phosphatidylcholines (PC), Alkylphosphatidylcholine (PC-O), Lysophosphatidylcholine (LPC), Alkyllysophosphatidylcholine (LPC-O), phosphatidylethanolamine (PE), Alkyl phosphatidylethanolamine (PE-O), Lysophosphatidylethanolamine (LPE), Alkyllysophosphatidylethanolamine (LPE-O), Phosphatidylglycerol (PG), Phosphatidylinositol (PI), Phosphatidylserine (PS), Phosphatic acid (PA) (Supplementary Table S1). Identified lipid profiles were compared using both multivariate and univariate statistics. In the principal component analysis (PCA) plot, the analyzed samples clustered into five distinct groups (Figure 2B). The growth media (GM) group, comprising undifferentiated cells, and the differentiated media (DM) group, which is differentiated cells, were clearly separated. The WHS treatment group showed a lipid profile close to that of the GM group in a concentration-dependent manner, even though differentiation was induced. Notably, the lipid profile of the WHS 200 µg/mL treatment group was observed to be almost the same level as that of the GM group. To identify the lipid class responsible for these differences, we confirmed the variations among each lipid class. In the DM group, a significant increase in TG and TG-O was characteristically noted compared to the GM group. Treatment with 200 µg/mL of WHS effectively suppressed this alteration in lipid metabolism (Figure 3A). Among the significant differences in the total abundance of each lipid class, glycerolipids including TG, TG-O, and DG were the most apparent, and were significantly reduced even in the group treated with the lowest concentration of 50 µg/mL (Figure 3B). On the other hand, in sphingolipid metabolism, all subclasses including SM, Cer and HexCer increased significantly in the DM group; however, a significant decrease was observed in the Cer and HexCer at WHS 200 µg/mL treatment (Figure 3C). Overall glycerophospholipids showed significant upregulation according to 3T3-L1 cell differentiation, and among these, concentration-dependent inhibition of WHS treatment was observed only in PC, LPC-O, PE, PE-O, LPE, LPE-O, PA, PG, and PI (Figure 3D).

Univariate analysis was performed to identify lipid markers of the inhibitory effect of adipogenesis and WHS treatment. In the DM group, a total of 541 lipid species showed a significant difference of *p* < 0.05 compared to the GM group. The changes in a significant number of lipids by differentiation were suppressed by WHS treatment, and at higher concentrations, much more lipid species appeared at a level close to the GM group. The details are provided in Supplementary Table S2.

### 3.3. Effect of WHS on Glycerolipid Metabolism during Adipogenesis in 3T3-L1 Preadipocyte

**Figure 4:**
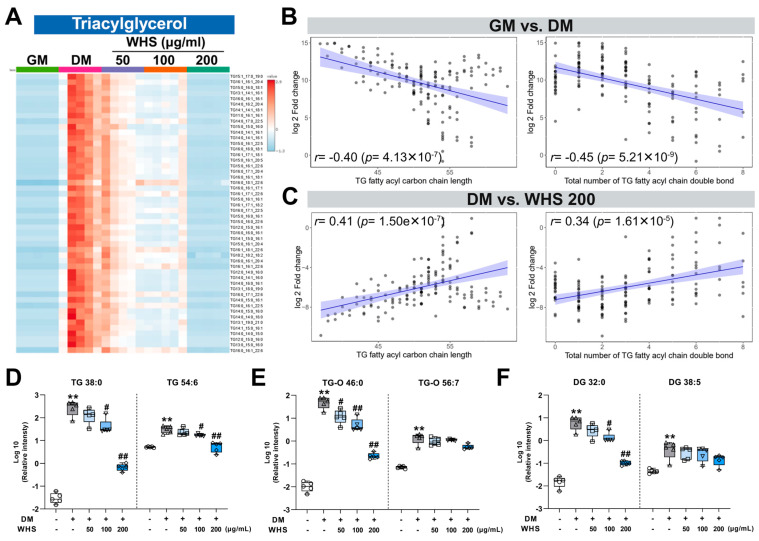
Alterations in glycerolipid levels upon WHS treatment during the differentiation of 3T3-L1 cells. (**A**) Heatmap analysis using the top 50 triacylglycerol (TG) species showing significant differences between sampling groups. Relative values are divided by the mean center and the standard deviation of each variable. Correlation with log2-fold change in total TG fatty acyl carbon chain length, double bond number, and TG molecular species (**B**) between GM and DM groups and (**C**) between DM and WHS 200 μg/mL treatment groups. Correlation plots were presented with each metabolite value (point) plotted along a linear regression line (blue) with a 95% confidence interval (blue area). The significant differences between sampling groups in molecular species of (**D**) triacylglycerol (TG), (**E**) alkyltriacylglycerol (TG-O), and (**F**) diacylglycerol (DG), composed of relatively short-chain saturated fatty acids and those composed of long-chain polyunsaturated fatty acids. GM denotes the growth media group (control); DM denotes the differentiation media group (differentiation); WHS denotes the differentiated group treated with WHS at 50, 100, 200 μg/mL. Graphs are presented as mean ± SEM (*n* = 5, ** *p* < 0.01 vs. GM; # *p* < 0.05 vs. DM; ## *p* < 0.01 vs. DM).

Consistent with previous findings, the TG levels were found at much higher levels after differentiation of 3T3-L1 cells than undifferentiated cells [21] (Figure 4A). In the same manner, in our results, TG-O and DG classes were also significantly increased like TG (Supplementary Figure S1A,B). These changes during adipogenesis are associated with increased activity of GPAT3, which has fatty-acyl-CoA catalytic activity, and AGPAT2, which catalyzes the second step of the glycerol phosphate pathway, the main pathway of TG synthesis [22]. In addition, DG level also changed in relation to the activity of DGAT, which catalyzes the production of DG [23]. The increase in most glycerolipids caused by differentiation was suppressed by WHS treatment and almost no increase was observed at a concentration of 200 µg/mL. This inhibitory effect of WHS could be related to the suppression of TG and DG production due to transcriptional upregulation during adipogenesis, and the results demonstrated that the WHS effectively regulates the overall upregulation of glycerolipid metabolism leading to TG accumulation.

We also performed correlation analysis to investigate whether the degree of this change differs depending on the characteristics of the fatty acyl chain of TG (Supplementary Table S3). Interestingly, the fatty acyl carbon chain length and the total number of double bonds constituting TG showed a negative correlation with the fold change in TG molecular species during adipogenesis (Figure 4B). For example, TG 38:0, which is saturated and has a relatively short carbon chain length, increased to about 9677-fold higher in the DM group than in the GM group, while TG 54:6, which has a long carbon chain length and high degree of unsaturation, increased to only 6-fold higher (Figure 4D). These results implicate that the increases in saturated and short-chain TG species were more pronounced than that of long-chain and unsaturated species. On the contrary, the WHS 200 µg/mL treatment group showed a positive correlation in fold difference compared to DM, which confirms the increase in short-chain and saturated TG species was relatively more suppressed (Figure 4C). The inhibitory effect of WHS on DM-induced upregulation of TG was also much stronger in TG 38:0 than in TG 54:6, showing differences depending on the TG molecular species. These results indicate that the inhibition of changes in TG species by WHS treatment mainly affects short-chain and saturated TG species. An increase in saturated and short-chain fatty acids due to decreased activity of fatty acid desaturase and elongase in serum and tissues has been reported in metabolic syndrome and obesity [24,25]. Additionally, changes in TG fatty acid composition have been found to be correlated with insulin sensitivity, type 2 diabetes, and abdominal obesity [26,27]. In our study, changes in the composition of TG species seen after 3T3-L1 cell differentiation were consistent with those in obesity and related diseases, and these changes were effectively suppressed by WHS. These results confirmed that WHS does not simply reduce total TG content but also causes changes in the fatty acid composition of TG species, which has the potential to exert beneficial effects in alleviating obesity and related diseases. These differences in TG species composition were also observed in the TG-O and DG classes (Supplementary Figure S1C,D), at the intracellular level in TG-O 46:0 and TG-O 56:7, and DG 32:0 and DG 38:5 (Figure 4E,F). This confirms that there was a clear difference between groups and demonstrates that the pattern of changes in TG species composition due to adipogenesis affects overall glycerolipid metabolism and that WHS treatment effectively inhibits these changes.

### 3.4. Effect of WHS on Glycerophospholipid Metabolism during Adipogenesis in 3T3-L1 Preadipocyte

**Figure 5:**
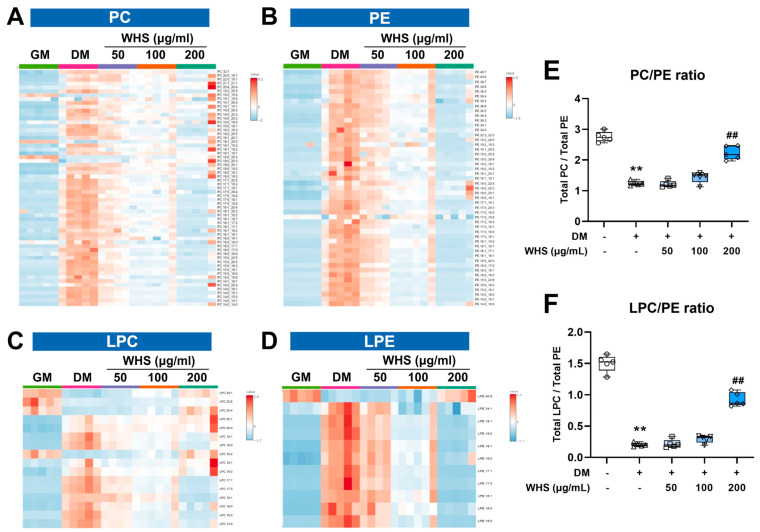
Alterations in glycerophospholipid levels upon WHS treatment during the differentiation of 3T3-L1 cells. Heatmap analysis using (**A**) phosphatidylcholine (PC), (**B**) phosphatidylethanolamine (PE), (**C**) lysophosphatidylcholine (LPC), and (**D**) lysophosphatidylethanolamine (LPE) species showing significant differences between sampling groups. Relative values are divided by the mean center and the standard deviation of each variable. (**E**) PC/PE ratio and (**F**) LPC/PE ratio in 3T3-L1 cells. GM denotes the growth media group (control); DM denotes the differentiation media group (differentiation); WHS denotes the differentiated group treated with WHS at 50, 100, 200 μg/mL. Graphs are presented as mean ± SEM (*n* = 5, ** *p* < 0.01 vs. GM; ## *p* < 0.01 vs. DM).

Phospholipids, including PC, PE, PS, etc., are the main constituents of animal cell membranes, serving as a matrix supporting membrane proteins, signaling molecules in response to external stimuli, and immune monitoring [28,29]. Phospholipids also maintain lipid metabolic homeostasis by playing a role in the assembly of lipoproteins required for TG and cholesterol transport, and thus they are crucial metabolites in biological systems, including lipid metabolism [30]. In particular, PC and PE are the main phospholipids of mammalian cell membranes. They are distributed asymmetrically in the outer leaflet and inner leaflet and maintain membrane integrity. Therefore, disruption of the homeostasis of these two lipid classes can cause changes in protein function [31]. LPC and LPE, the fundamental components of cellular membranes, can be generated from PC and PE by the partial hydrolysis via phospholipase A2 (PLA2) [32]. These are most abundant glycerophospholipids in biological samples and play an important role in inflammatory diseases by altering various functions in immune cells [33]. Our findings indicate that the total abundance of phospholipid subclass changes increased during the differentiation of 3T3-L1 cells (Figure 3D). The most abundant changes in molecular species were observed in PC, PE, LPC, and LPE with the trends varying by specific molecular species following differentiation or WHS treatment (Figure 5A–D, Supplementary Table S2). Since this difference may be attributed to the effect of fatty acyl composition, a correlation analysis was conducted. Consequently, the number of carbon chains in fatty acyls showed positive and negative correlations with the changes observed in PC, PE, and LPE after differentiation and WHS 200 µg/mL treatment, respectively (Supplementary Figure S2). Additionally, the number of double bonds in fatty acyls significantly correlated with the changes observed in PC and LPE. An example of the alteration in levels of molecular species composed of short-chain and saturated fatty acids, as well as those composed of long-chain and unsaturated fatty acids, due to differentiation and WHS treatment, is shown in Supplementary Figure S3. These changes in the composition of molecular species in PC and PE may result from alterations in fatty acid composition due to the inhibition of elongase and desaturase during lipogenesis, as well as impacts on glycerolipid metabolism. However, these changes occurring in PC and PE lead to alterations in the relative amounts of these two lipids in the membrane, which play a crucial role in maintaining homeostasis and can significantly impact membrane integrity and immunity [34].

Changes in the PC/PE ratio have been reported as a marker that affects various biological functions and as a critical modulator of various diseases [35]. The decrease in the PC/PE ratio may be due to a lack of phosphatidylethanolamine N-ethyltransferase (PEMT), an enzyme that partially methylates PE to generate PC [36]. A decreased level of the PC/PE ratio can negatively affect energy metabolism and insulin sensitivity and can increase the accumulation of lipid droplets. This change becomes a major factor in mitochondrial dysfunction and inflammatory processes and has been reported in nonalcoholic fatty liver disease (NAFLD) and hepatic steatosis [37]. In our results, the significant decrease in the PC/PE ratio during the adipogenesis may be related to the accumulation of lipid droplets, and these changes were suppressed by WHS treatment (Figure 5E). Inhibition of these changes may result in metabolic changes that affect not only lipid accumulation but also risk factors for inflammation and liver disease. On the other hand, the decrease in the LPC/PE ratio due to an increase in PE showed a stronger correlation with NAFLD progression than the PC/PE ratio reported previously [38]. The LPC/PE ratio was also showed in the same pattern as the PC/PE ratio in our results (Figure 5F). Overall, WHS effectively defended against imbalance in the composition and ratio of PC and PE caused by adipogenesis, and the 200 µg/mL treatment group showed an inhibitory effect at an almost undifferentiated level. This result suggests that the effect of WHS on glycerophospholipid metabolism is related to fat accumulation. In addition, it suggests the possibility of reducing risk factors for obesity-related comorbidities such as NAFLD and diabetes.

### 3.5. Effect of WHS on Sphingolipid Metabolism during Adipogenesis in 3T3-L1 Preadipocyte

Sphingolipids are structural lipids and signaling molecules whose homeostasis is maintained through regulated de novo synthesis and degradation [39]. Among sphingolipids, Cer serves as the main precursor for the synthesis of other sphingolipids. The biosynthesis of CER is initiated by serine palmitoyltransferase (SPT), using serine and palmitoyl-CoA as substrates to sequentially produce ketosphinganine, dihydrosphingosine, and dihydroceramide. The generated Cer can be metabolized through the hydrolytic, catabolic, and sphingomyelin pathway, among which the SM pathway is a crucial pathway for producing SM, the most abundant sphingolipid [40]. The upregulation of genes involved in Cer biosynthesis and the subsequent increase in SM and Cer levels in obesity and metabolic syndrome were reported [41,42]. Furthermore, significantly increased expression of several enzymes (sptlc2, sgms1, and CerS6) involved in the de novo synthesis of Cer was reported in diet-induced insulin resistant mice [43]. This improvement in insulin sensitivity and weight loss through inhibition of Cer metabolism upregulation has been suggested as an effective treatment target for obesity and T2DM [44].

**Figure 6:**
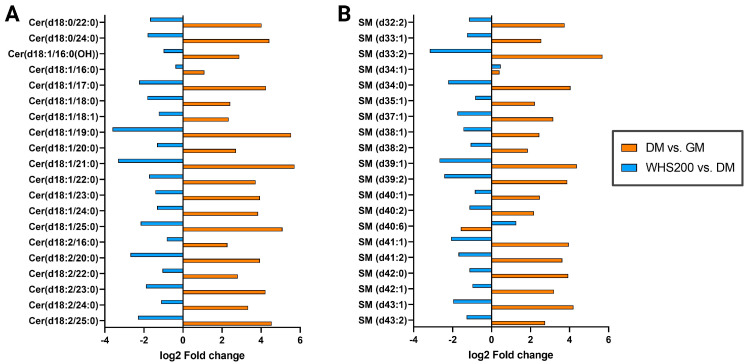
Alterations in sphingolipid levels upon WHS treatment during the differentiation of 3T3-L1 cells. The Log2-fold changes in the top 20 (**A**) Cer and (**B**) SM showing significant differences between sampling groups. The orange bar represents the changes during differentiation, and blue bar represents the differences between WHS-treated (200 μg/mL) group and non-treated group. GM denotes the growth media group (control); DM denotes the differentiation media group (differentiation); WHS 200 denotes the differentiated group treated with WHS at 200 μg/mL.

Our results also showed that the total abundance of SM, Cer, and HexCer increased after differentiation, consistent with previous reports [9] (Figure 3C). In the WHS treatment group, the increase in Cer and HexCer due to differentiation was significantly suppressed, but no significant change was observed in SM. This result arises because not all SM species showed the same trend of change, and these differences were not correlated with the characteristics of the fatty acyl chain, such as carbon chain length and number of double bonds (Supplementary Table S3). However, the top 20 ceramide (Cer) and sphingomyelin (SM) species that showed significant differences due to WHS treatment effectively inhibited the upregulation of sphingolipids in 3T3-L1 cells caused by differentiation, following the same trends (Figure 6). The inhibition of Cer and SM upregulation may result from WHS effectively suppressing the Cer biosynthesis induced by differentiation, thereby preventing the elevation of sphingolipids. It has been shown that this upregulation of sphingolipids is associated with improvement not only in obesity but also in inflammation and hepatic steatosis [45,46]. Therefore, this suggests that prevention of imbalance in the sphingolipid metabolic pathway due to differentiation by WHS treatment could be an effective preventive target for clinical markers related to obesity and metabolic diseases.

## 4. Conclusions

**Figure 7:**
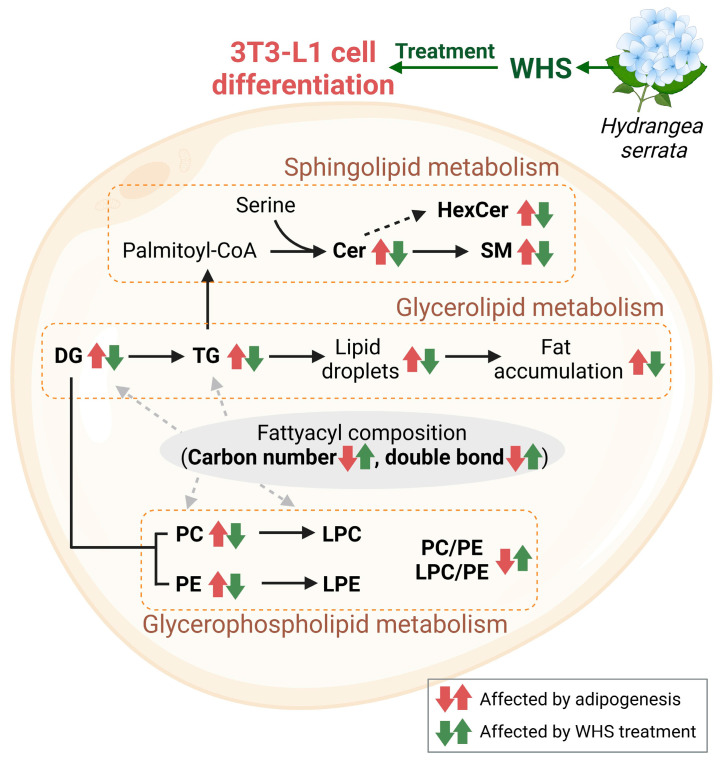
Overview of lipid metabolic pathways changed by WHS treatment during the differentiation of 3T3-L1 cells. The significantly changed lipids are indicated by arrows. Red arrows represent changes induced by adipogenesis, and green arrows indicate changes by WHS treatment. Lipids include Ceramide (Cer), Hexosylceramide (HexCer), Sphingomyelin (SM), Diacylglycerol (DG), Triacylglycerol (TG), Phosphatidylcholine (PC), Phosphatidylethanolamine (PE), Lysophosphatidylcholine (LPC), and Lysophosphatidylethanolamine (LPE).

In conclusion, our study demonstrated that treatment with WHS significantly inhibits changes in lipid metabolism associated with adipogenesis in 3T3-L1 preadipocytes (Figure 7). Specifically, WHS treatment not only effectively suppresses the upregulation of total glycerolipids but also alters the fatty acyl composition, shifting from short-chain saturated fatty acids to long-chain unsaturated fatty acids, leading to metabolic benefits. This molecular shift also influences glycerophospholipid metabolism throughout, and WHS effectively prevents the differentiation-derived imbalance in glycerophospholipids, which play a crucial role in maintaining cell membrane stability and metabolic homeostasis. Moreover, it effectively regulates the PC/PE and LPC/PE ratios, which are markers of NAFLD, inflammation, and insulin resistance. In sphingolipid metabolism, WHS inhibits the levels of Cer and SM, both of which are elevated in obesity and related metabolic diseases. Consequently, this study comprehensively evaluated the overall lipid metabolic mechanisms within preadipocyte cells to explore the systemic effects of WHS on adipogenesis. Changes in the lipidomic profiles induced by WHS not only improve insulin sensitivity, membrane integrity, and immunity-related disorders, but also positively affect lipid markers. This contributes to reducing risk factors for obesity and its associated comorbidities, such as type 2 diabetes mellitus (T2DM) and fatty liver disease. This study is the first to assess the metabolic mechanisms of WHS’s adipogenesis-inhibitory effects through changes in the composition of lipid molecular species and various lipid markers. Our findings provide valuable insights into the development of nutraceuticals or therapeutic application for a better understanding and management obesity-related metabolic disorders.

## Data Availability

The original contributions presented in the study are included in the article, further inquiries can be directed to the corresponding authors.

## Supplementary Materials

The following supporting information can be downloaded at: https://www.mdpi.com/article/10.3390/nu16101508/s1, Figure S1: Alterations in TG-O and DG upon WHS treatment during the differentiation of 3T3-L1 cells. (A) Heatmap analysis of TG-O and (B) DG. Relative values are divided by the mean center and the standard deviation of each variable. The correlation with the log2-fold change in total fatty acyl carbon chain length and double bond number Comparisons include (C) TG-O and (D) DG molecular species between GM and DM groups, as well as between DM and WHS 200 μg/mL treatment groups, Figure S2: The correlation between log2-fold change and total fatty acyl carbon chain length or double bond number in molecular species of (A) PC, (B) PE, (C) LPC, and (D) LPE, Figure S3: The significant differences between sampling groups in molecular species of (A) phosphatidylcholine (PC), (B) phosphatidylethanolamine (PE), (C) lysophosphatidylcholine (LPC), and (D) lysophosphatidylethanolamine (LPE), composed of relatively short-chain saturated fatty acids and those composed of long-chain polyunsaturated fatty acids, Table S1. Identified lipids in LC-MS-based lipidomics results. A total of 619 lipid species from 18 classes were detected in the positive and negative ion modes, Table S2. Potential lipid biomarker in 3T3L1 cells showed statistically significant difference among sampling group, Table S3. Correlation between fatty acyl carbon chain length, double bonds and fold changes in each lipid class using Spearman’s correlation coefficient.
