# Supplementary material for: Lipidomic Assessment of the Inhibitory Effect of Standardized Water Extract of Hydrangea serrata (Thunb.) Ser. Leaves during Adipogenesis
Source: Nutrients. 2024 May 16;16(10):1508. doi: 10.3390/nu16101508 (PMC11124303; doi:10.3390/nu16101508)
Supplement: Supplementary file 1 [file nutrients-16-01508-s001.zip › supplementary figures_revised.pdf]

Lipidomic assessment of the inhibitory effect of Standardized water extract of *Hydrangea serrata* (Thunb.) Ser. leaves Extract during adipogenesis

Jae Sik Yu[1,2], Hee Ju Kim[1,2], Yeo Eun Kim[1,2], Hyun Ok Yang[1,2], Yu-Kyong Shin[3], Hyunjae Kim[3], Soyoon Park[3], Gakyung Lee[1,2*]

[1] Department of Integrative Biological Sciences and Industry, Sejong University, Seoul 05006, Republic of Korea

[2] Convergence Research Center for Natural Products, Sejong University, Seoul 05006, Republic of Korea

[3] Department of New Material Development, COSMAXBIO, Seongnam 13486, Republic of Korea

* Corresponding author

Gakyung Lee, Department of Integrative Biological Sciences and Industry, and Convergence Research Center for Natural Products, Sejong University, 209, Neungdong-ro, Gwangjin-gu, Seoul, Republic of Korea

Tel.: +82-2-6935-2479

E-mail: lgg1025@sejong.ac.kr

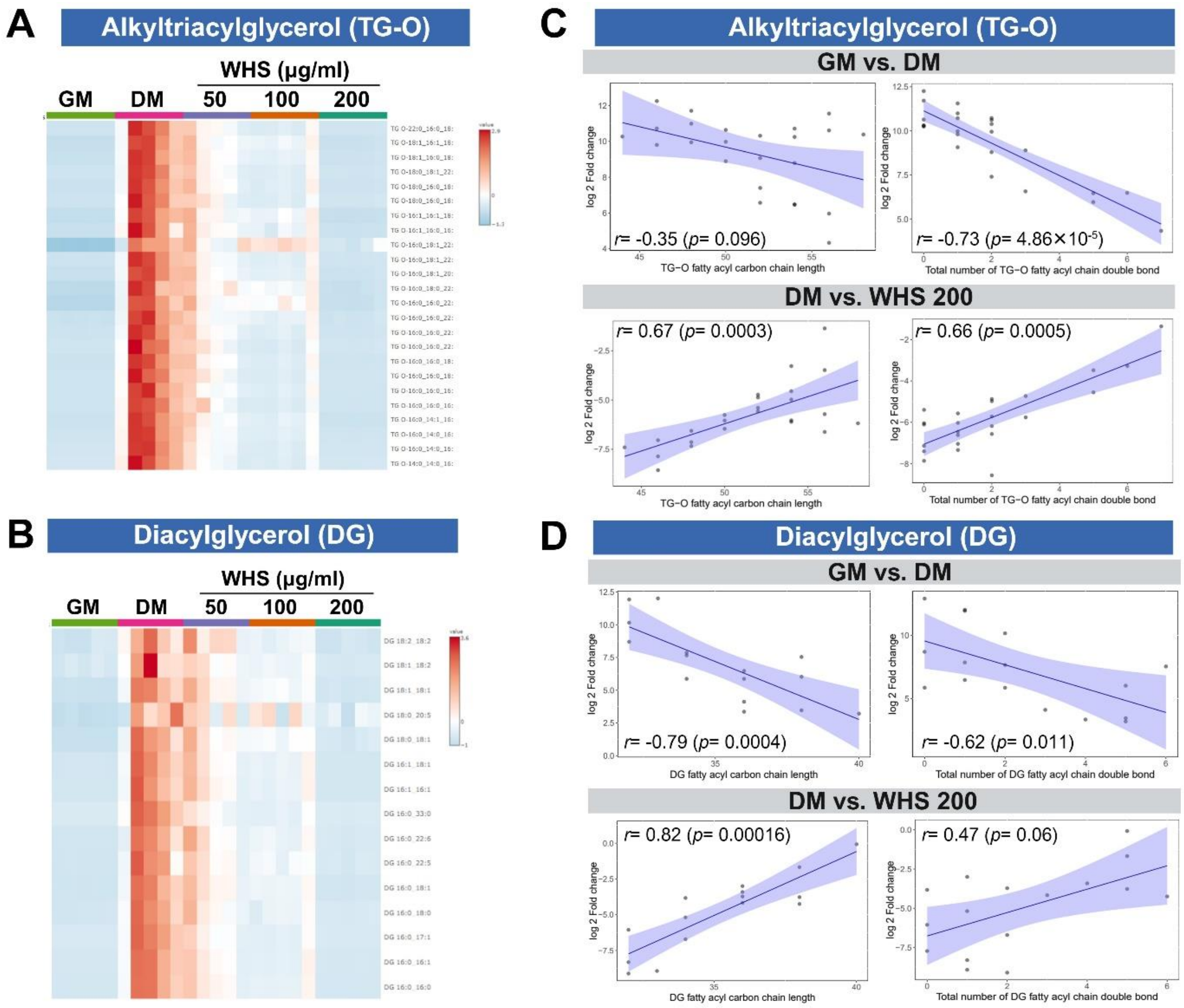

Supplementary Figure S1. Alterations in TG-O and DG upon WHS treatment during the differentiation of 3T3-L1 cells. (A) Heatmap analysis of TG-O and (B) DG. Relative values are divided by the mean center and the standard deviation of each variable. The correlation with the log2-fold change in total fatty acyl carbon chain length and double bond number Comparisons include (C) TG-O and (D) DG molecular species between GM and DM groups, as well as between DM and WHS 200 μg/mL treatment groups. Correlation plots were presented with each metabolite value (point) plotted along a linear regression line (blue) with a 95% confidence interval (blue area). GM denotes the growth media group (control); DM denotes the differentiation media group (differentiation); WHS denotes the differentiated group treated with WHS at 50, 100, 200 μg/mL.

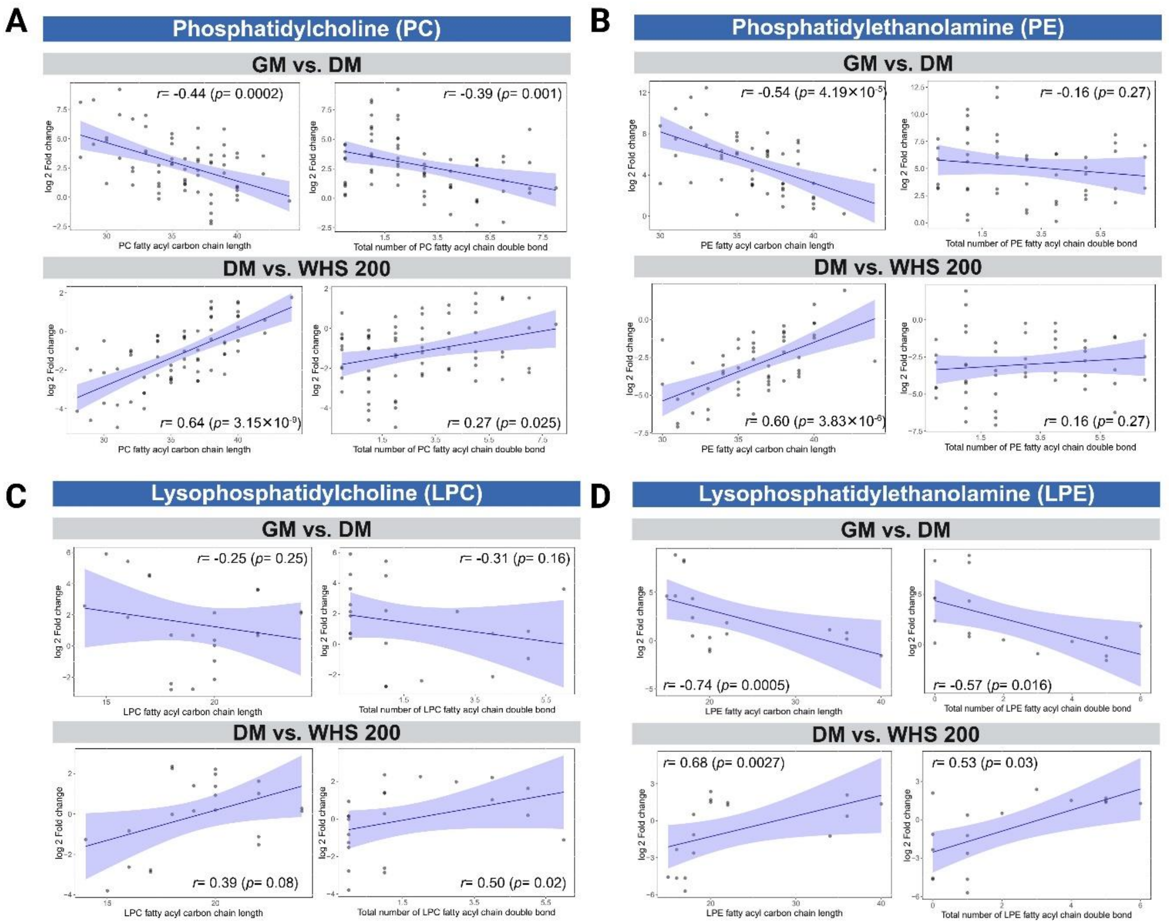

Supplementary Figure S2. The correlation between log2-fold change and total fatty acyl carbon chain length or double bond number in molecular species of (A) PC, (B) PE, (C) LPC, and (D) LPE. Correlation plots were presented with each metabolite value (point) plotted along a linear regression line (blue) with a 95% confidence interval (blue area). 'GM vs. DM' represents the fold change between the control group and the differentiation group. 'DM vs. WHS 200' represents the fold change between the differentiated group and the WHS 200 µg/mL treatment group. GM denotes the growth media group (control); DM denotes the differentiation media group (differentiation); WHS 200 denotes the differentiated group treated with WHS at 200 µg/mL.

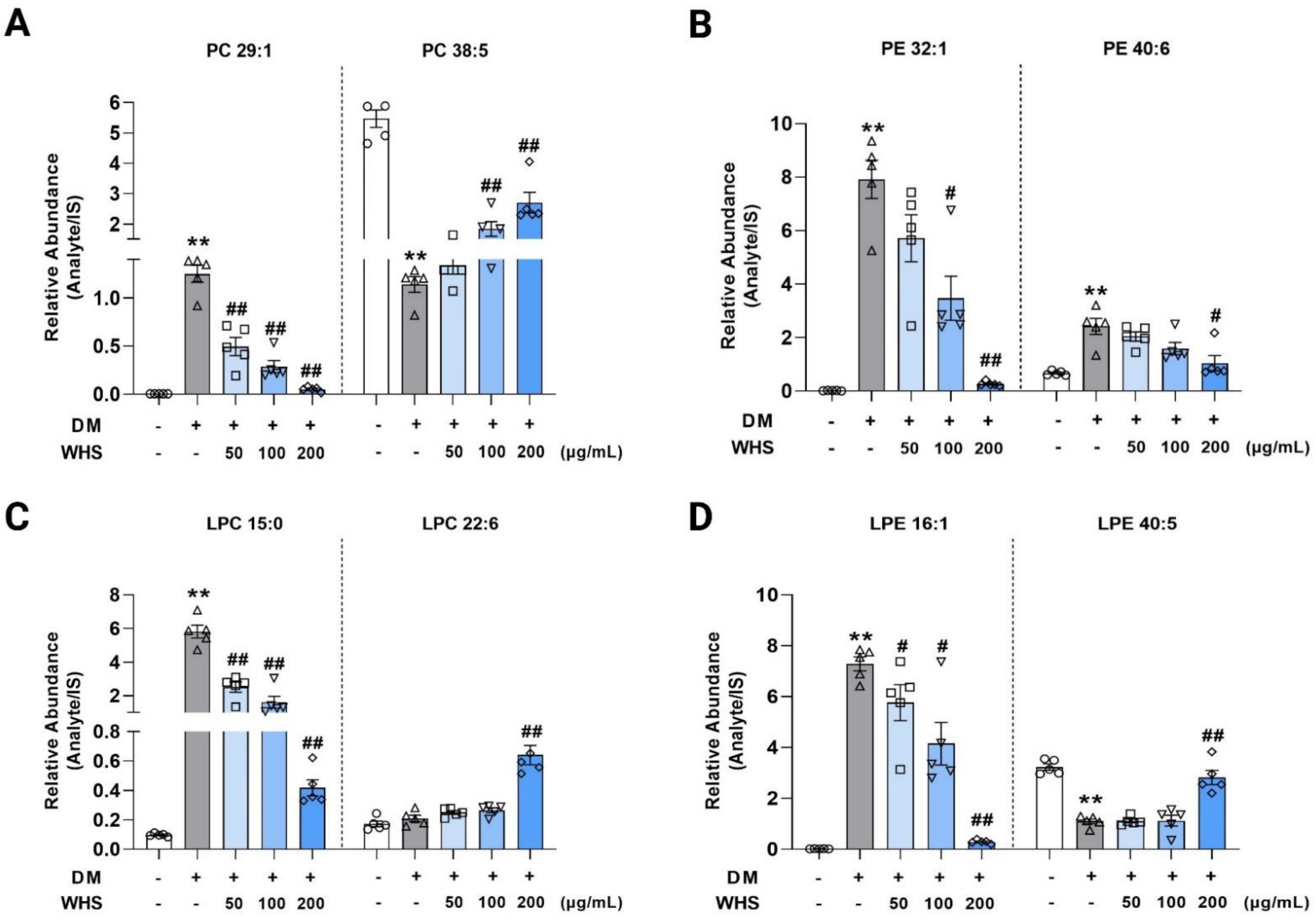

Supplementary Figure S3. The significant differences between sampling groups in molecular species of (A) phosphatidylcholine (PC), (B) phosphatidylethanolamine (PE), (C) lysophosphatidylcholine (LPC), and (D) lysophosphatidylethanolamine (LPE), composed of relatively short-chain saturated fatty acids and those composed of long-chain polyunsaturated fatty acids. DM denotes the differentiation media group (differentiation); WHS denotes the differentiated group treated with WHS at 50, 100, 200 μg/mL. Graphs are presented as mean ± SEM (n=5, **p < 0.01 vs.GM; #p < 0.05 vs. DM; ##p < 0.01 vs. DM).
